# A Recombinant Chimeric Protein-Based Vaccine Containing T-Cell Epitopes from Amastigote Proteins and Combined with Distinct Adjuvants, Induces Immunogenicity and Protection against *Leishmania infantum* Infection

**DOI:** 10.3390/vaccines10071146

**Published:** 2022-07-19

**Authors:** Daniela P. Lage, Danniele L. Vale, Flávia P. Linhares, Camila S. Freitas, Amanda S. Machado, Jamille M. O. Cardoso, Daysiane de Oliveira, Nathália C. Galvani, Marcelo P. de Oliveira, João A. Oliveira-da-Silva, Fernanda F. Ramos, Grasiele S. V. Tavares, Fernanda Ludolf, Raquel S. Bandeira, Isabela A. G. Pereira, Miguel A. Chávez-Fumagalli, Bruno M. Roatt, Ricardo A. Machado-de-Ávila, Myron Christodoulides, Eduardo A. F. Coelho, Vívian T. Martins

**Affiliations:** 1 Programa de Pós-Graduação em Ciências da Saúde: Infectologia e Medicina Tropical, Faculdade de Medicina, Universidade Federal de Minas Gerais, Av. Prof. Alfredo Balena, 190, Belo Horizonte 30130-100, MG, Brazil; danipagliara@hotmail.com (D.P.L.); dani.dlv@hotmail.com (D.L.V.); flaviaprata13@hotmail.com (F.P.L.); camilasimoesf@gmail.com (C.S.F.); manda_sanchez92@hotmail.com (A.S.M.); nathaliagalvani05@gmail.com (N.C.G.); marcelloperdigao@outlook.com (M.P.d.O.); joaoaosilva@gmail.com (J.A.O.-d.-S.); fe.fonsecaramos@gmail.com (F.F.R.); grasysv@hotmail.com (G.S.V.T.); feludolf@gmail.com (F.L.); raquelsoares.id@gmail.com (R.S.B.); amorim.gpereira@gmail.com (I.A.G.P.); eduardoferrazcoelho@yahoo.com.br (E.A.F.C.); viviantamietti@yahoo.com.br (V.T.M.); 2 Laboratório de Imunopatologia, Núcleo de Pesquisas em Ciências Biológicas (NUPEB), Departamento de Ciências Biológicas, Insituto de Ciências Exatas e Biológicas, Universidade Federal de Ouro Preto, Ouro Preto CEP 35400-000, MG, Brazil; jam.mirelle@gmail.com (J.M.O.C.); bmroatt@gmail.com (B.M.R.); 3 Programa de Pós-Graduação em Ciências da Saúde, Universidade do Extremo Sul Catarinense, Criciúma 88806-000, SC, Brazil; daysiolv@yahoo.com.br (D.d.O.); r_andrez@yahoo.com.br (R.A.M.-d.-Á.); 4 Computational Biology and Chemistry Research Group, Vicerrectorado de Investigación, Universidad Católica de Santa María, Urb. San José S/N, Umacollo, Arequipa 04000, Peru; mchavezf@ucsm.edu.pe; 5 Neisseria Research Group, Molecular Microbiology, Faculty of Medicine, School of Clinical and Experimental Sciences, University of Southampton, Southampton General Hospital, Southampton SO16 6YD, UK; 6 Departamento de Patologia Clínica, Colégio Técnico (COLTEC), Universidade Federal de Minas Gerais, Av. Antônio Carlos, 6627, Belo Horizonte 31270-901, MG, Brazil

**Keywords:** visceral leishmaniasis, vaccine, T-cell epitopes, polypeptide-based protein, immune response, adjuvants

## Abstract

Currently, there is no licensed vaccine to protect against human visceral leishmaniasis (VL), a potentially fatal disease caused by infection with *Leishmania* parasites. In the current study, a recombinant chimeric protein ChimT was developed based on T-cell epitopes identified from the immunogenic *Leishmania* amastigote proteins LiHyp1, LiHyV, LiHyC and LiHyG. ChimT was associated with the adjuvants saponin (Sap) or monophosphoryl lipid A (MPLA) and used to immunize mice, and their immunogenicity and protective efficacy were evaluated. Both ChimT/Sap and ChimT/MPLA induced the development of a specific Th1-type immune response, with significantly high levels of IFN-γ, IL-2, IL-12, TNF-α and GM-CSF cytokines produced by CD4^+^ and CD8^+^ T cell subtypes (*p* < 0.05), with correspondingly low production of anti-leishmanial IL-4 and IL-10 cytokines. Significantly increased (*p* < 0.05) levels of nitrite, a proxy for nitric oxide, and IFN-γ expression (*p* < 0.05) were detected in stimulated spleen cell cultures from immunized and infected mice, as was significant production of parasite-specific IgG2a isotype antibodies. Significant reductions in the parasite load in the internal organs of the immunized and infected mice (*p* < 0.05) were quantified with a limiting dilution technique and quantitative PCR and correlated with the immunological findings. ChimT/MPLA showed marginally superior immunogenicity than ChimT/Sap, and although this was not statistically significant (*p* > 0.05), ChimT/MPLA was preferred since ChimT/Sap induced transient edema in the inoculation site. ChimT also induced high IFN-γ and low IL-10 levels from human PBMCs isolated from healthy individuals and from VL-treated patients. In conclusion, the experimental T-cell multi-epitope amastigote stage *Leishmania* vaccine administered with adjuvants appears to be a promising vaccine candidate to protect against VL.

## 1. Introduction

Leishmaniasis is a parasitic disease complex caused by distinct *Leishmania* species and is considered one of the six high-priority tropical diseases, with 12 million people clinically affected in over 98 countries and 380 million people exposed to the risks of infection annually [1]. There are two main clinical manifestations: tegumentary leishmaniasis (TL), which is the most common form of leishmaniasis causing significant patient morbidity, and visceral leishmaniasis (VL), which is a life-threatening disease condition affecting the patients’ organs, e.g., the spleen, liver and bone marrow [2,3]. TL is caused by several parasite species such as *Leishmania braziliensis*, *L. amazonensis*, *L. panamensis*, *L. guyanensis*, *L. mexicana*, *L. aethiopica*, *L. major* and *L. tropica*, whereas VL is caused by the *L. infantum* and *L. donovani* species [4,5].

Preventative measures against VL include the control and/or elimination of reservoir vectors, including barriers to sand fly feeding using residual sprays, treated netting/clothing, topical repellents and/or applications in reservoir burrows [6]. There is also the precise diagnosis and rapid treatment of infections of humans and dogs, since VL is a zoonotic disease with a canine reservoir [7,8]. However, such measures are insufficient to prevent disease spread within endemic countries, and, in this context, prophylactic vaccination is considered a promising approach to induce long-term and cost-effective protection in mammalian hosts [9]. However, a human vaccine does not exist, and the few available canine vaccines demonstrate variable efficacy in endemic countries, adverse effects, and declining long-term protection, among other attributes.

It is generally considered that a Th1-type immune response is required for protection against *Leishmania*, with the production of cytokines, such as interferon (IFN)-γ, interleukin (IL)-12, tumor necrosis factor (TNF)-α and granulocyte-macrophage colony-stimulating factor (GM-CSF), among others, required to stimulate parasitized macrophages to produce reactive oxygen species that promote parasite death [10,11]. Conversely, a Th2-type response characterized by the production of cytokines, such as IL-4, IL-10 and IL-13, among others, deactivates parasitized macrophages and allows the disease to progress [12]. Thus, candidate leishmanial vaccines should stimulate a specific Th1-type response in immunized mammalian hosts.

Many distinct VL vaccine candidates have been tested in murine and/or canine models [13,14,15,16], including plasmid DNA-based [17,18] and recombinant protein-based vaccines [19,20]. However, few have progressed to human trials, and the use of individual *Leishmania* proteins as recombinant molecules limits the antigenic repertoire of the experimental vaccines. Polypeptide-based vaccines, on the other hand, have certain advantages, since they can contain several T-cell epitopes from distinct parasite immunogenic proteins to hypothetically induce a more robust Th1-type response in vaccinees [21,22]. Indeed, several experimental studies have shown the efficacy of such vaccines to protect against VL in animal models [23,24,25].

Previously, we showed that a recombinant chimeric protein, composed of specific CD4^+^ and CD8^+^ T-cell epitopes from distinct *Leishmania* proteins that recognized mouse and human haplotypes, induced a Th1-type response in immunized mice that provided protection against *L. infantum* infection [26]. More recently, we developed a chimeric protein named ChimeraT that included T-cell epitopes from four *Leishmania* proteins (prohibitin, eukaryotic initiation factor 5a and two hypothetical proteins). Immunization of mice with adjuvanted ChimeraT stimulated a Th1-type specific immune response that protected mice against *L. infantum* infection. Higher production of IFN-γ, IL-12 and GM-CSF cytokines by both murine T-cell subtypes, with correspondingly low levels of IL-4 and IL-10 cytokines, were detected [25].

Promising new VL vaccines should eventually be evaluated in humans, e.g., in the recently established challenge model [27], or, at the very least, with ex vivo stimulation experiments with human immune cells, followed by evaluation of Th1-type cytokine production [28]. In addition, most of the tested vaccine candidates have been developed with promastigote stage proteins. However, it has been hypothesized that amastigote protein antigens may be more appropriate for vaccine development as this parasite stage remains in contact with the host immune system during active disease [29,30]. Based on this hypothesis, in the present study we evaluated four amastigote stage *Leishmania infantum* proteins (LiHyp1, LiHyV, LiHyC and LiHyG) previously shown to be immunogenic and protective in a murine model of infection by bioinformatics tools that predict the main T-cell epitopes to construct a gene encoding a chimeric protein. The chimeric protein (named ChimT) was administered alone or associated with two immune adjuvants, saponin and monophosphoryl lipid A (MPLA), and the vaccine immunogenicity and protective efficacy against VL were evaluated in BALB/c mice. In addition, ChimT was used to stimulate human peripheral blood mononuclear cells (PBMCs) from healthy individuals and taken from non-treated or treated VL patients, and cytokines were evaluated in culture supernatants after in vitro stimulation.

## 2. Material and Methods

### 2.1. Blood Samples

The present study was approved by the Ethics Committee on Human Research of Federal University of Minas Gerais (UFMG, Belo Horizonte, Minas Gerais, Brazil), with protocol number CAAE–32343114.9.0000.5149. Peripheral blood samples (20 mL) were collected from VL patients (*n* = 6; 2 male and 4 female, with ages ranging from 29 to 53 years), before and 6 months after treatment using pentavalent antimonials (Sanofi Aventis Farmacêutica Ltd.a, Suzano, São Paulo, Brazil). Infection was confirmed by polymerase chain reaction (PCR) technique targeting *L. infantum* kDNA in spleen and/or bone marrow aspirates from the patients. Samples were also collected from healthy individuals living in an endemic area of VL (Belo Horizonte; *n* = 6; 2 male and 4 female, with ages ranging from 34 to 50 years) who had no signs of leishmaniasis and had negative serological results with the Kalazar Detect™ Test (InBios International^®^, Seattle, WA, USA).

### 2.2. Parasite and Mice

*L. infantum* MHOM/BR/1970/BH46 stationary promastigotes were grown at 24 °C in complete Schneider’s medium (Sigma-Aldrich, St. Louis, MO, USA), which was composed of medium plus 20% (*v*/*v*) heat-inactivated fetal bovine serum (FBS, Sigma-Aldrich), 20 mM L-glutamine, 100 U/mL penicillin and 50 µg/mL streptomycin, pH 7.4. The Soluble *Leishmania* Antigenic (SLA) extract was prepared as described previously [31]. BALB/c mice (female, 8 weeks of age) were obtained from Bioterism Center, UFMG and were maintained under specific pathogen-free conditions. The study was approved by the Committee on the Ethical Handling of Research Animals from UFMG, with protocol number 144/2020.

### 2.3. Construction of ChimT Protein

The main T-cell epitopes from proteins LiHyp1 (XP_001468941.1), LiHyV (XP_001462854.1), LiHyC (XP_001470432.1) and LiHyG (XP_001467126.1) were predicted by bioinformatics, and their nucleotide sequences were used to construct the gene encoding the chimeric protein. CD4^+^ and CD8^+^ T-cell epitopes were evaluated by Rankpep server [32], choosing A2, A3, A24 and B7 alleles for human CD8^+^ T cells (MHC-I) and H-2Db, H-2Dd, H-2Kb, H-2Kd, H-2Kk and H-2Ld alleles for mouse CD8^+^ T cells (MHC-I). For the selection of CD4^+^ T-cell epitopes, the HLA-DR1, HLA-DR2, HLA-DR3, HLA-DR4, HLA-DR5, HLA-DR8, HLA-DR9, HLA-DR11, HLA-DR12, HLA-DR13 and HLA-15 alleles were used for human CD4^+^ T cells (MHC-II), while the H-2IAb, H-2IAd, H-2IAs, H-2IEd and H-2IEb alleles were used for mouse CD4^+^ T cells (MHC-II). The binding threshold parameters used were 2% and 5% for MHC-I and MHC-II, respectively. B-cell epitopes were also predicted in the amino acid sequences of four proteins, and they were excluded in the sequence from the chimeric protein. The main regions containing human and mouse-specific T-cell epitopes were selected and used to construct the chimeric protein. The arrangement of epitopes in the protein sequence was chosen to mimic their arrangement in the native protein. The protein sequence was submitted for selection using specific codons to optimize expression in *Escherichia coli* cells with the web codon optimization tool (https://www.idtdna.com/CodonOpt). The MFOLD Program was used to reduce the presence of intramolecular interactions of mRNA. To avoid spatial overlap between T-cell epitopes, two glycine (GLY) residues and three lysine (LYS) residues were included between the epitopes, and they were also added at the proximal and terminal portions of the chimeric protein sequence to increase solubility. The physical–chemical characteristics of ChimT were evaluated with the ProtParam tool in the ExPASy server.

### 2.4. Purification of Recombinant ChimT Protein

The gene encoding ChimT was commercially synthesized in the pET28a-TEV vector by GenScript^®^ (Piscataway, NJ, USA). The recombinant protein was expressed in *E. coli* Artic Express cells (DE3, Agilent Technologies, Santa Clara, CA, USA) adding 1 mM of isopropyl β-D-1-thiogalactopyranoside (IPTG; Sigma-Aldrich, St. Louis, MO, USA), with shaking at 100 rpm for 24 h at 12 °C. The bacterial cells were ruptured by five cycles of ultrasonication of 30 s. each (38 MHz) followed by six cycles of freezing and thawing. Debris was removed by centrifugation and ChimT was passed over a HisTrap HP affinity column connected to an AKTA system (GE Healthcare, Boston, MA, USA) and further purified on a Superdex™ 200 gel-filtration column (GE Healthcare Life Sciences, Boston, MA, USA). The purified protein was then passed over a polymyxin-agarose column (Sigma-Aldrich, St. Louis, MO, USA) to remove any residual endotoxin content: less than 10 ng of lipopolysaccharide per 1 mg of protein was detected with the Quantitative Chromogenic Limulus Amebocyte Kit (QCL-1000 model, BioWhittaker, Walkersville, MD, USA) following the manufacturer’s instructions.

### 2.5. Mouse Immunization and Experimental Infection

BALB/c mice (*n* = 16 per group) were vaccinated subcutaneously in their left, hind footpad with ChimT (20 μg) alone or associated with saponin (20 μg; *Quillaja saponaria* bark saponin, Sigma-Aldrich, St. Louis, MO, USA) or monophosphoryl lipid A (MPLA, 20 μg; catalog 1246298-63-4, Sigma-Aldrich, St. Louis, MO, USA) in a volume of 50µL per footpad. In addition, control animals received saponin (20 μg), MPLA (20 μg) or saline only. In the six experimental groups, animals received three doses of the products, which were administered at 14-day intervals, and 30 days after the last dose they (*n* = 8 per group) were euthanized, and their spleens and sera were collected for immunological assays. At the same time, the remaining animals (*n* = 8 per group) were infected subcutaneously in the right, hind footpad with 10^7^ *L. infantum* stationary promastigotes, and 45 days post-infection they were euthanized and their spleens, draining lymph nodes and bone marrow removed and sera collected for immunological and parasitological assays [28].

### 2.6. Cellular Response

#### 2.6.1. Cytokine and Nitrite Production and Evaluation of T-Cell Subtypes Producing IFN-γ

Splenocytes were obtained from animals before and after challenge (*n* = 8 per group, in each step), and cells (5 × 10^6^ per well) were cultured in complete RPMI 1640 medium (control), which was composed by medium plus 20% (*v*/*v*) FBS, 20 mM L-glutamine, 200 U/mL penicillin and 100 µg/mL streptomycin, pH 7.4, or stimulated with ChimT or *L. infantum* SLA (10.0 and 25.0 μg/mL, respectively), for 48 h at 37 °C in 5% (*v*/*v*) CO_2_. Commercial capture ELISA kits (BD OptEIA TM set mouse kits, Pharmingen, San Diego, CA, USA) were used to evaluate levels of IFN-γ, IL-4, IL-10, IL-12 and GM-CSF cytokines in the culture supernatants, following the manufacturer’s instructions. In addition, cell supernatants from infected and immunized animals were used to evaluate nitrite production using the Griess reaction, as described previously [25]. To evaluate the participation of CD4^+^ and CD8^+^ T-cell subtypes in producing IFN-γ secretion after challenge, spleen cells were incubated in the presence of monoclonal antibodies against mouse CD4 (GK 1.5) or CD8 (53-6.7) molecules (5 μg/mL each) for 48 h at 37 °C in 5% (*v*/*v*) CO_2_. The cell supernatants were collected, and IFN-γ production was evaluated by capture ELISA. Appropriate isotype-matched controls—rat IgG2a (R35-95) and rat IgG2b (95-1)—were used, and all antibodies (no azide/low endotoxin) were purchased from BD Pharmingen (San Diego, CA, USA) [19].

#### 2.6.2. IFN-γ Expression in the Infected and Immunized Mice

IFN-γ gene expression was evaluated after challenge in the SLA-stimulated splenocyte cultures by RT-qPCR technique [28]. RNA was extracted from mouse spleens (*n* = 8 per group) by using TRIzol reagent (Invitrogen, Carlsbad, CA, USA) following the manufacturer’s instructions. It was suspended in UltraPure™ DNase/RNase-free distilled water (Invitrogen, Carlsbad, CA, USA), and RNA concentration was measured with a NanoDrop LITE spectrophotometer (Thermo Scientific, Waltham, MA, USA) by using the λ260/280 nm absorbance ratios. Sample quality was evaluated with agarose (1.5% *w*/*v*) electrophoresis gel. The extracted RNA was treated for 15 min at room temperature with DNAse I (Invitrogen, Carlsbad, CA, USA) and the enzyme was deactivated using 25 mM of EDTA for 10 min at 65 °C. Total RNA (2 μg) was reverse transcribed using Applied Biosystems High-Capacity cDNA Reverse Transcription Kit (Thermo Scientific, Carlsbad, CA, USA), forming complementary deoxyribonucleic acid (cDNA) by using the parameters of 25 °C for 10 min, 37 °C for 120 min, and 85 °C for 5 min. RT-qPCR was performed using Applied Biosystems PowerUp™ SYBR™ Green PCR master mix (Thermo Scientific, Carlsbad, CA, USA) and gene-specific primers for IFN-γ (*Forward* 5′-TCAAGTGGCATAGATGTGGAAGAA-3′ and *Reverse* 5′-TGGCTCTGCAGGATTTTCATG-3′) in a 7900HT Thermocycler (Applied Biosystems, Waltham, MA, USA). Transcripts were normalized using ACTB and GAPDH housekeeping genes. The procedure was optimized by adjusting the primer concentrations to 5, 10 and 15 pmol to determine optimal specificity and efficiency. The material purity was verified by melting curves and gel electrophoresis. The cycle parameters were an initial denaturation at 95 °C for 10 min, followed by 40 cycles at 95 °C for 15 s, and annealing/extension at 60 °C for 1 min, followed by a dissociation stage for recording the melting curve. Results were shown graphically as the fold changes in gene expression by using the mean ± standard deviation of target gene [28]. Data were analyzed according to relative expression using the 2^−ΔΔCT^ method.

#### 2.6.3. Polyfunctional T-Cell Analyses by Flow Cytometry

In vitro procedures for labeling intracytoplasmic cytokines were performed as described previously [22] and consisted primarily of immunostaining cell surface markers, followed by intracellular cytokine staining. Briefly, splenocytes were obtained by maceration of spleens harvested from the animals under sterile conditions and incubated in complete RPMI medium in 96-well round-bottom culture plates at a concentration of 5 × 10^5^ cells per well. Cultured cells were non-stimulated (control) or stimulated with SLA (25 μg/mL) and incubated for 48 h at 37 °C in 5% (*v*/*v*) CO_2_. Brefeldin A (Sigma-Aldrich, USA) was added at a final concentration of 10 μg/mL, and the cultures were incubated under the same conditions for an additional 4 h. Some wells were stimulated with Phorbol 12-myristate 13-acetate (PMA-5 ng/mL) and ionomycin (1 μg/mL) as positive controls. Afterwards, cells were labeled with Fixable Viability Stain 450 (FVS450, BD Biosciences, San Diego, CA, USA) for 15 min at room temperature followed by staining with antibodies against CD3 (BV650 anti-mouse, clone 145.2C11), CD4 (BV605 anti-mouse, clone RM4-5) and CD8 (BV786 anti-mouse, clone 53-6.7) at room temperature for 30 min. Cells were fixed with FACS fixing solution, washed and permeabilized with PBS buffer plus 0.5% (*w*/*v*) saponin and stained with antibodies against IL-2 (PE anti-mouse, clone JES6-5H4), IFN-γ (AF700 anti-mouse, clone XMG1.2), TNF-α (PE-Cy7 anti-mouse, clone LG.3A10) and IL-10 (APC anti-mouse, clone JES5-16E) at room temperature for 30 min. All antibodies were purchased from BD Biosciences (San Diego, CA, USA). Samples were read on a LSR Fortessa cytometer (BD Biosciences, San Diego, CA, USA) in which 100,000 events were acquired. Final data of intracytoplasmic cytokine production assay were expressed as indices, calculated by dividing the percentage of positive cells observed in the SLA-stimulated culture by the percentage observed in paired control, unstimulated culture (SLA/CC).

#### 2.6.4. In Vitro Splenocyte Proliferation

A lymphoproliferation assay was performed using spleen cells from infected and immunized mice (*n* = 8 per group). For this, splenocytes (5 × 10^6^ cells per well) were cultured in 96-well plates and non-stimulated (medium) or stimulated with ChimT or SLA (10.0 and 25.0 μg/mL, respectively) for 24 h at 37 °C in 5% (*v*/*v*) CO_2_. Next, 3-(4,5-dimethylthiazol-2-yl)-2,5-diphenyltetrazolium bromide (MTT; 10 μL of 5 mg/mL; Sigma-Aldrich, USA) was added to the wells followed by incubation for 4 h at 37 °C in 5% (*v*/*v*) CO_2_. After discarding the supernatant, intracellular formazan crystals were dissolved in 200 µL dimethyl sulfoxide (DMSO, Sigma-Aldrich, USA), and cell proliferation was evaluated in a spectrophotometer, at λ = 492 nm [28].

### 2.7. Analysis of IgG Production and Isotype Profile

Antibody production specific for ChimT and SLA was evaluated before and after challenge (*n* = 8 per group, in each step) by collecting sera from animals as described previously [25]. Titration curves were prepared to determine the most appropriate concentration of antigens and serum sample dilution to be used. Thus, immunoassay microplates (Jetbiofil^®^, Belo Horizonte) were coated with ChimT or SLA (0.5 and 1.0 µg per well, respectively), which were diluted in coating buffer (50 mM carbonate buffer at pH 9.6) for 18 h at 4 °C. Free binding sites were blocked using 250 µL of PBS plus 0.05% (*v*/*v*) Tween 20 (PBS-T) plus 5% (*w*/*v*) bovine serum albumin (BSA) for 1 h at 37 °C. After washing plates five times with PBS-T, wells were incubated with sera (1/100 diluted in PBS-T) for 1 h at 37 °C. Wells were again washed five times with PBS-T and incubated with peroxidase-labeled antibodies specific to mouse IgG, IgG1 and IgG2a (all diluted 1/10,000 in PBS-T; Sigma-Aldrich, USA), for 1 h at 37 °C. After washing the wells five times with PBS-T, reactions were developed through incubation with H_2_O_2_, ortho-phenylenediamine and citrate-phosphate buffer at pH 5.0 for 30 min in the dark and stopped by adding 25 µL of 2 N H_2_SO_4_. The optical density (OD) values were read in a microplate spectrophotometer (Molecular Devices, Spectra Max Plus, San Jose, CA, USA) at λ = 492 nm.

### 2.8. Parasite Load

#### 2.8.1. Limiting Dilution Technique

Organ parasitism was evaluated by a limiting dilution technique in the infected and immunized animals (*n* = 8 per group). For this, spleens, livers, bone marrow and draining lymph nodes were collected from mice, weighed and homogenized separately with glass tissue grinders in sterile PBS. Debris was removed by centrifugation at 150× *g* and cells were concentrated by centrifugation at 2000× *g*. Pellets were suspended in 1 mL of complete Schneider’s medium and 220 µL were plated into 96-well flat-bottom microtiter plates (Nunc) and diluted in log-fold serial dilutions using complete medium (10^−1^ to 10^−12^ dilution). Each sample was plated in triplicate and incubated at 24 °C and read 7 days after the beginning of culture. Results were expressed as the negative log of the titer through dilution corresponding to the last positive well, which was adjusted per milligram of organ [26].

#### 2.8.2. qPCR Assay

Splenic parasitism was evaluated by qPCR technique as described recently [28]. Spleen DNA was extracted using the Wizard^®^ Genomic DNA purification kit (Promega Corporation), following the manufacturer’s instructions. The resulting DNA was suspended in 100 µL of milli-Q H_2_O, and parasite was detected using *Forward* (CCTATTTTACACCAACCCCCAGT) and *Reverse* (GGGTAGGGGCGTTCTGCGAAA) primers. Mouse β-actin gene was used as an endogenous control to normalize nucleated cells and to verify sample integrity. Standard curves were obtained from DNA extracted from 10^8^ parasites for kDNA and 10^8^ peritoneal macrophages for β-actin. PCR was performed with a StepOne™ Instrument (48 wells-plate; Applied Biosystems) using 2× SYBR™ Select Master Mix (5 µL; Applied Biosystems), with 2 mM of each primer (1 µL) and 4 µL of DNA (25 ng/µL). Samples were incubated for 10 min at 95 °C and submitted to 40 cycles of 95 °C for 15 s and 60 °C for 1 min, and, during each time, fluorescence data were collected. Parasite quantification for each spleen sample was calculated by interpolation from the standard curve, performed in duplicate, and converted into the number of parasites per nucleated cell.

### 2.9. Immunogenicity Stimulation in Human Cells

ChimT-induced immunogenicity was evaluated in human cells and for this procedure blood samples were collected from VL patients (*n* = 6, obtained before and after treatment) as well as from healthy individuals (*n* = 6), and PBMCs were purified as described elsewhere [18]. PBMCs (10^7^ per well) were cultured in 48-well flat-bottomed tissue culture plates (Costar, Cambridge, MA, USA) in RPMI medium (control) or stimulated with ChimT or SLA (10 and 25 µg/mL, respectively) for 5 days at 37 °C in 5% (*v*/*v*) CO_2_. Culture supernatants were collected and the levels of IFN-γ and IL-10 cytokines were measured by capture ELISA using commercial kits (Human IFN-γ and IL-10 ELISA Sets, BD Biosciences, San Diego, CA, USA), according to the manufacturer’s instructions.

### 2.10. Statistical Analysis

Data were entered into Microsoft Excel (version 10.0) spreadsheets and analyzed using GraphPad Prism™ (version 6.0 for Windows). Statistical analysis was one-way analysis of variance (ANOVA) followed by Bonferroni´s post-test, which was used to compare between the groups. The immunization experiments were performed twice, and all in vitro assays were performed at least twice, and results are representative. Differences were considered significant when *p* values were <0.05.

## 3. Results

### 3.1. Construction and Characterization of Recombinant Chimeric Protein, ChimT

The amino acid sequences of *Leishmania* amastigote stage proteins LiHyp1, LiHyV, LiHyC and LiHyG were evaluated with bioinformatics tools to predict the main CD4^+^ and CD8^+^ T-cell epitopes, which were then used to construct a chimeric protein termed ChimT. Our analyses identified the epitope YIMSGPARYVYFHMVLPVEAQ in the LiHyp1 sequence, the epitope GVCVANTNVAAGAHTAALAAAVCVV epitope in the LiHyV sequence, the epitope LLFVNQKLVGTIADVRSYEK in the LiHyC sequence and the epitope SLFVLYMYVTCRGGYTYLQL in the LiHyG sequence. The ChimT amino acid sequence is shown in Table 1 along with the physical–chemical characteristics of the recombinant protein. ChimT was predicted to be a soluble and stable recombinant protein.

### 3.2. ChimT Plus Adjuvant Stimulates the Development of a Th1-Type Cellular Response before and after Infection

**Figure 1:**
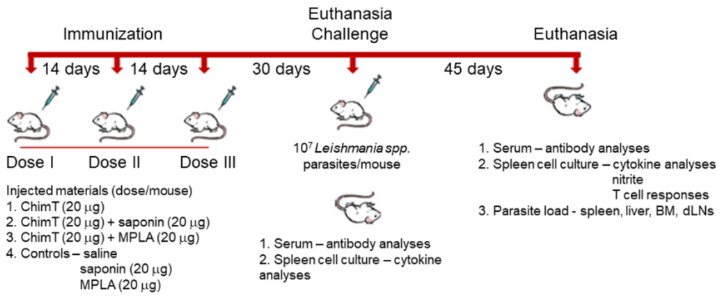
**A flowchart for the experimental protocol to examine the immunogenicity of ChimT in mice.**

**Figure 2:**
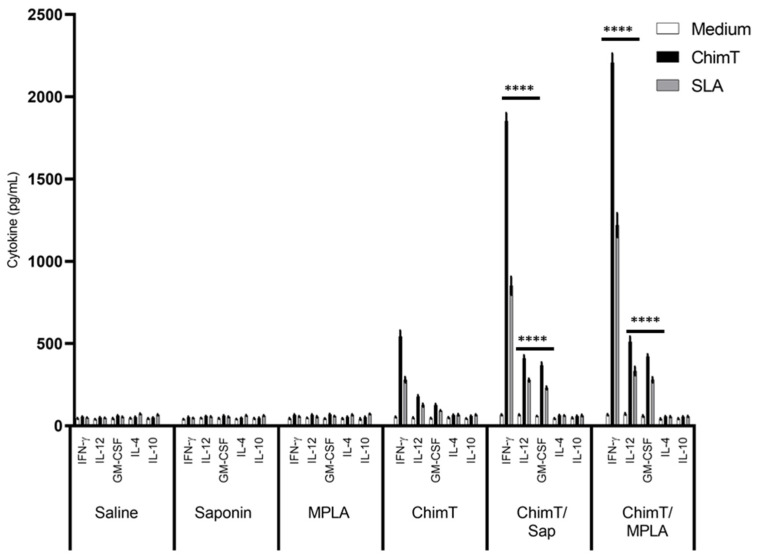
**Cytokine production before *L. infantum* infection.** Mice (*n* = 8 per group) received saline or were immunized with saponin, MPLA, ChimT, ChimT/Sap or ChimT/MPLA. Thirty days after the last vaccine dose, they were euthanized and their spleen cells (5 × 10^6^ cells per mL) were cultured in DMEM and non-stimulated (medium) or stimulated with ChimT or SLA (10 and 25 µg/mL, respectively) for 48 h at 37 °C in 5% (*v*/*v*) CO_2_. The cell supernatant was collected and levels of IFN-γ, IL-12, GM-CSF, IL-4 and IL-10 were measured by capture ELISA. Bars indicate the mean ± standard deviation of groups. (****) indicates significant difference in relation to the saline, saponin, MPLA and ChimT groups (*p* < 0.00001).

A flowchart for the immunization, challenge, euthanasia and sampling protocol to examine the murine immune response to experimental *Leishmania* vaccines is shown in Figure 1. BALB/c mice were immunized with ChimT alone or with ChimT and the adjuvants saponin (ChimT/Sap group) or MPLA (ChimT/MPLA group). Control mice received saline, saponin or MPLA alone. Initially, the immune response was evaluated in in vitro cell culture supernatants of splenocytes removed from immunized mice before infection and stimulated with ChimT, SLA or medium alone. Culture supernatants from splenocytes from mice immunized with ChimT/Sap and ChimT/MPLA and stimulated with ChimT and SLA had similar and significantly higher levels of IFN-γ, IL-12 and GM-CSF cytokines when compared to the control groups, and they had correspondingly low levels of IL-4 and IL-10 (Figure 2). Mice immunized with ChimT alone (i.e., no adjuvant) had detectable levels of IFN-γ, IL-12 and GM-CSF, but these were significantly lower than the responses observed with the adjuvant groups.

**Figure 3:**
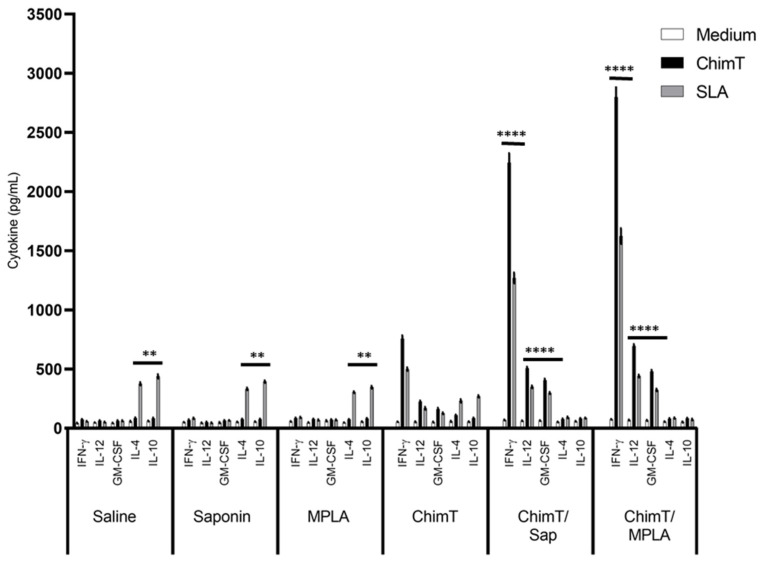
**Cellular response developed after challenge.** Mice (*n* = 8 per group) received saline or were immunized with saponin, MPLA, ChimT, ChimT/Sap or ChimT/MPLA. Thirty days after the last vaccine dose, they were infected with *L. infantum* promastigotes, and 45 days post-challenge, their spleen cells (5 × 10^6^ cells per mL) were cultured in DMEM and non-stimulated (medium) or stimulated with ChimT or SLA (10 and 25 µg/mL, respectively) for 48 h at 37 °C in 5% (*v*/*v*) CO_2_. The cell supernatant was collected and levels of IFN-γ, IL-12, GM-CSF, IL-4 and IL-10 were also measured by capture ELISA. Bars indicate the mean ± standard deviation of groups. (****) indicates significant difference in relation to the saline, saponin, MPLA and ChimT groups (*p* < 0.00001). (**) indicates significant difference in relation to the ChimT/Sap and ChimT/MPLA groups (*p* < 0.001).

Groups of immunized mice were also infected with live *L. infantum* parasites, and the immune response was evaluated (Figure 3). The cellular profile was sustained in the ChimT/Sap- and ChimT/MPLA-immunized and -infected mice, with increased levels of IFN-γ, IL-12 and GM-CSF when compared to the uninfected mice (Figure 2). The cytokine levels from the mice immunized with ChimT alone and then infected did not increase substantially over the levels observed in uninfected mice (Figure 2). Comparing the vaccinated groups for both immunized mice (Figure 2) and immunized and infected mice (Figure 3), although the cytokine levels were higher for the ChimT/MPLA groups compared to the ChimT/Sap groups, they were statistically similar. Again, no significant production of these cytokines was observed in the control groups (Figure 3), although these now showed significantly higher levels of antileishmanial IL-4 and IL-10 cytokines (Figure 3).

**Figure 4:**
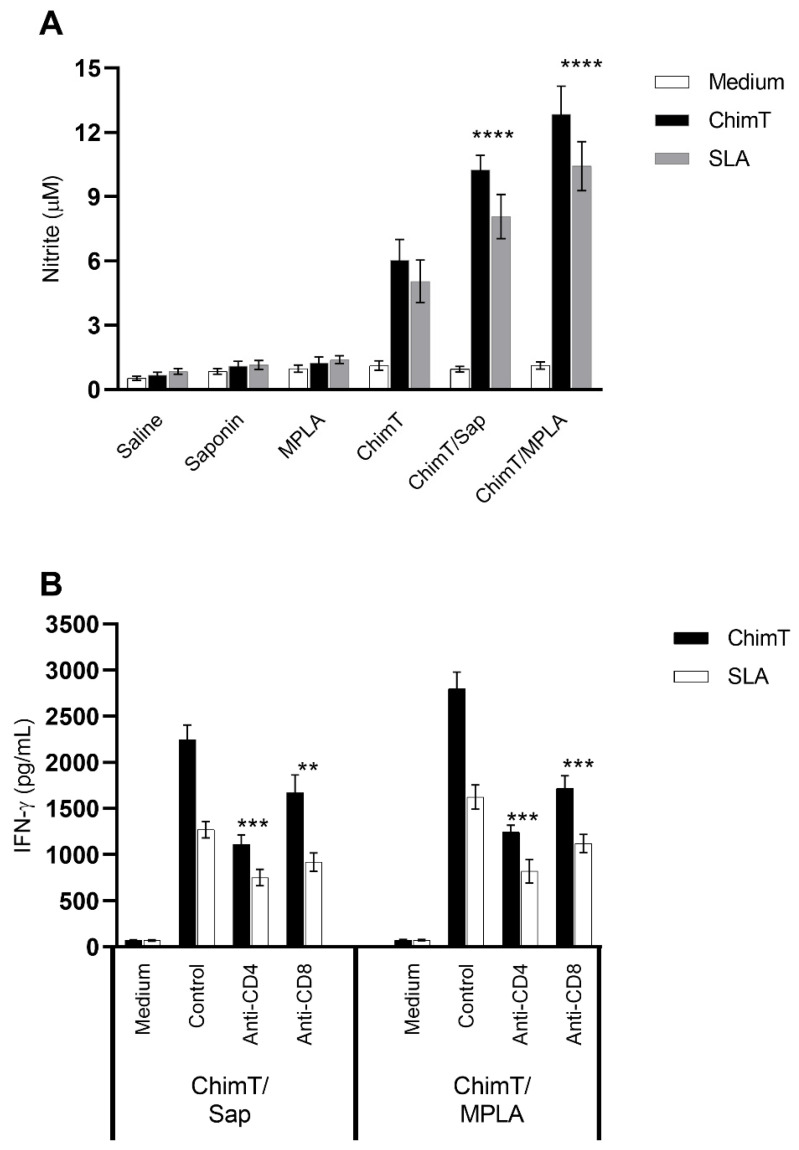
**Involvement of CD4^+^ and CD8^+^ T cell subtypes in the (A) nitrite and (B) IFN-γ pro-duction production after infection.** Mice (*n* = 8 per group) were vaccinated and later challenged with *L. infantum* promastigotes. Forty-five days post-infection, their spleen cells (5 × 10^6^ cells per mL) were cultured in DMEM and non-stimulated (medium) or stimulated with ChimT or SLA (10.0 and 25.0 μg/mL, respectively) for 48 h at 37 °C in 5% (*v*/*v*) CO_2_. In some wells, anti-CD4 or anti-CD8 monoclonal antibodies were added in the cultures (5 µg/mL each). In those, the cell supernatant was collected, and IFN-γ production was also measured by ELISA capture. (****) indicates significant difference in relation to the saline, saponin, MPLA and ChimT groups (*p* < 0.00001). In addition, in the wells without the addition of monoclonal antibodies, supernatants were collected, and nitrite secretion was evaluated by Griess reaction. Bars indicate the mean ± standard deviation of groups. (**) and (***) indicate statistically significant difference in relation to the control cell cultures (incubated without monoclonal antibody) (*p* < 0.001 and *p* < 0.0001, respectively).

Nitrite production was also evaluated in the cell supernatants of cell cultures derived from the spleens of immunized and infected mice (Figure 4A). Cultures from mice immunized with ChimT/Sap and ChimT/MPLA and then infected, produced significantly higher levels of this antileishmanial molecule after splenocytes were stimulated with ChimT or SLA, when compared to the controls (Figure 4A). These levels were significantly higher than the positive nitrite production observed with the ChimT alone group.

**Figure 5:**
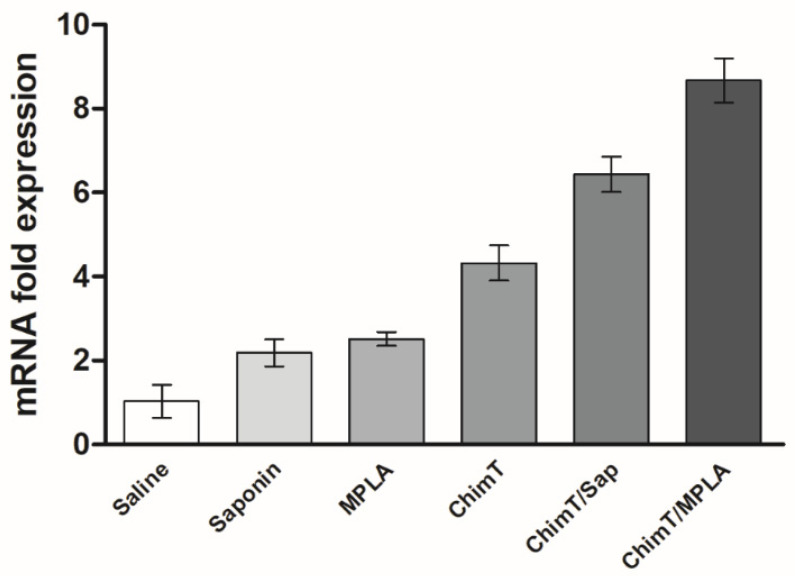
**IFN-γ mRNA expression after challenge infection.** Mice (*n* = 8 per group) received saline or were immunized with saponin, MPLA, ChimT, ChimT/Sap or ChimT/MPLA. Then, they were challenged with *L. infantum* promastigotes, and 45 days post-infection, their spleen cells (5 × 10^6^ cells per well) were stimulated with SLA (25.0 μg/mL) for 48 h at 37 °C in 5% (*v*/*v*) CO_2_. Cells were collected and mRNA was obtained and used to evaluate IFN-γ expression by qRT-qPCR. Data were normalized with control primers from housekeeping genes ACTB and GAPDH. Bars indicate the mean ± standard deviation of groups. Significant difference was observed for both ChimT/Sap and ChimT/MPLA over the saline, saponin, MPLA and ChimT groups with *p* < 0.00001. ChimT/MPLA showed significant difference over ChimT/Sap group with *p* < 0.05.

We also examined indirectly the participation of CD4^+^ and CD8^+^ T-cell subtypes in the production of IFN-γ production in the culture supernatants of splenocytes from immunized and infected mice (Figure 4B) after treatment with ChimT or SLA in vitro. The addition of anti-CD4^+^ and anti-CD8^+^ antibodies resulted in approximately two-fold statistically significant reductions in IFN-γ secretion for both ChimT/Sap and ChimT/MPLA immunized and infected mice, with a marginally greater reduction of cytokine secretion observed with anti-CD4^+^ antibody (Figure 4B). Activation of the cellular response was additionally investigated in the infected and vaccinated mice by means of detection of IFN-γ mRNA expression in stimulated spleen cell cultures using a RT-qPCR technique. Spleen cell cultures from mice immunized with ChimT/Sap and ChimT/MPLA and then stimulated with SLA expressed significantly three- to four-fold higher levels of IFN-γ, when compared to the values obtained in the control groups (Figure 5). Immunization with ChimT alone induced an approximately two-fold increase in mRNA expression compared to the controls (Figure 5).

**Figure 6:**
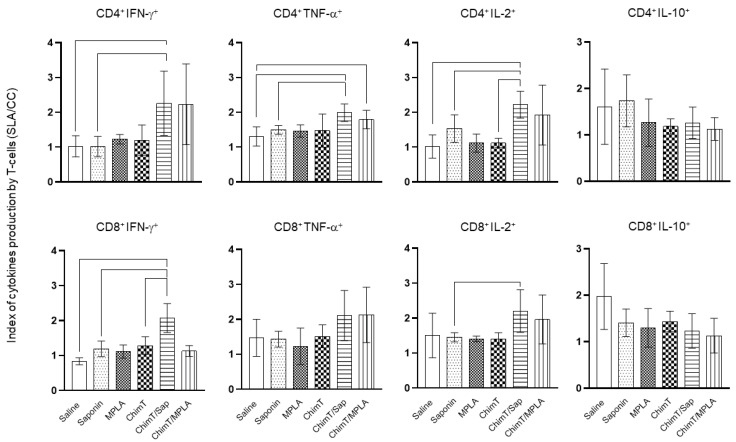
**Intracytoplasmic cytokine-producing T-cell profile evaluated by flow cytometry.** IFN-γ, TNF-α, IL-2 and IL-10-producing CD4^+^ and CD8^+^ T-cell subtypes were evaluated by the ratio between the frequency from stimulated versus non-stimulated cultures. The IFN-γ, TNF-α, IL-2 and IL-10-producing CD4^+^ and CD8^+^ T-cell percentages are shown. Bars indicate the mean plus standard deviation of groups. Lines between experimental groups indicate significant difference between them (*p* < 0.05).

**Figure 7:**
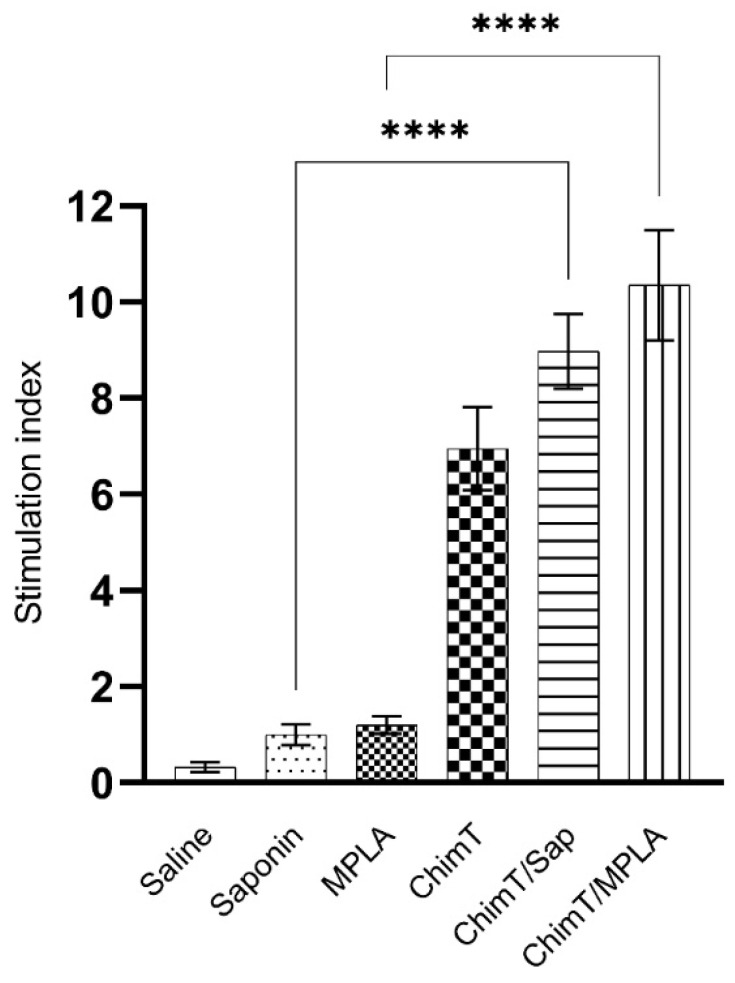
**Cellular proliferationevaluated after infection.** Spleen cells (5 × 10^6^ cells per well) were obtained from infected and vaccinated mice (*n* = 8 per group), and they were cultured in complete RPMI 1640 medium and stimulated with SLA (25.0 μg/mL) for 24 h at 37 °C in 5% (*v*/*v*) CO_2_. Cellular lymphoproliferation was evaluated by the MTT method. Bars indicate the mean ± standard deviation of groups. (****) indicates significant difference in relation to the indicated groups by lines (*p* < 0.00001).

A flow cytometry assay was performed with the SLA-stimulated spleen cells to evaluate the frequency of IFN-γ, TNF-α, IL-2 and IL-10-producing T cells (Figure 6). The key observations from these experiments were that ChimT/Sap and ChimT/MPLA immunized and infected mice had significantly increased indices of IFN-γ-producing CD4^+^ T cells and marginally higher but still statistically significant indices for TNF-α and IL-2 producing CD4^+^ T cells when compared to control groups. With respect to CD8^+^ T cells, significance was observed only for ChimT/Sap indices for IFN-γ and IL-2 production (Figure 6). A higher frequency of IL-10-producing CD4^+^ and CD8^+^ T cells was observed with stimulated spleen cell cultures from control mice, though not statistically significant to the indices for the ChimT immunized and infected mice. Representative plots of the gating strategy used to evaluate IFN-γ, TNF-α, IL-2 and IL-10 producing T cells by flow cytometry are shown in Supplementary Figure S1. A spleen cell lymphoproliferation assay was also performed after infection, and results showed that higher lymphoproliferative responses were observed with SLA-stimulated spleen cells from ChimT, ChimT/Sap and ChimT/MPLA mice compared to controls (Figure 7). Overall, a marginally higher Th1-type polarized immune response was observed with ChimT/MPLA vaccine compared to ChimT/Sap vaccine, although it was not statistically significant.

### 3.3. ChimT Plus Adjuvant Stimulates Specific IgG2a Isotype Production before and after Parasite Challenge

**Figure 8:**
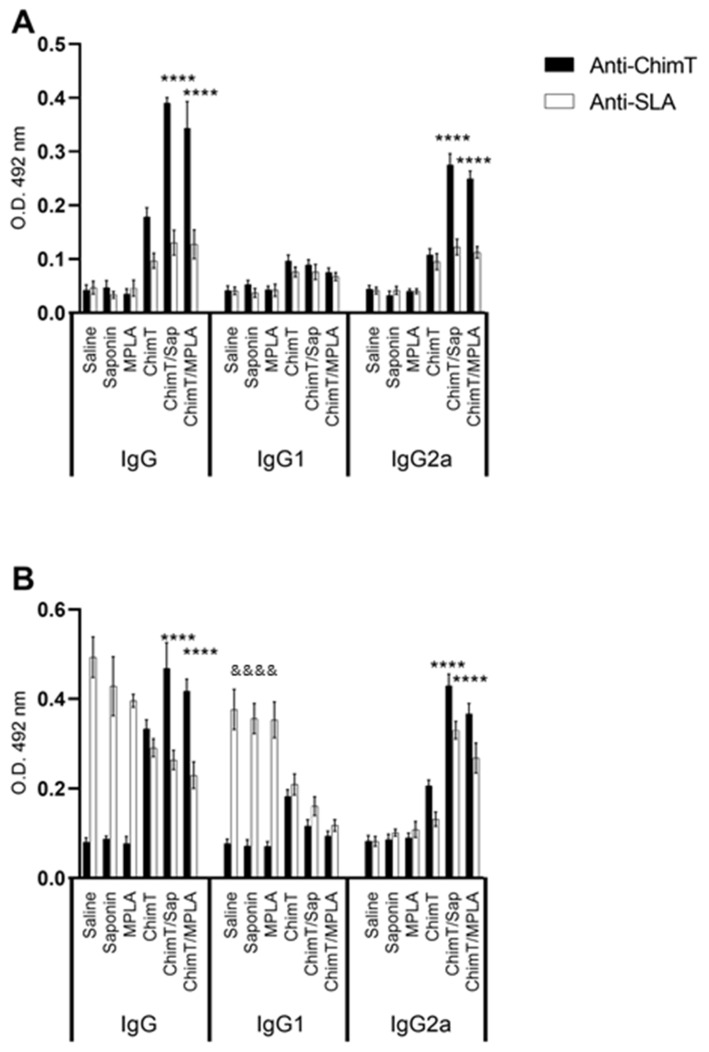
**Antibody response (A) before and (B) after challenge.** Mice (*n* = 8 per group) were vaccinated and later challenged with *L. infantum* promastigotes. Forty-five days post-infection, they were euthanized, and their sera samples were collected to evaluate the anti-ChimT and anti-SLA IgG1 and IgG2a levels by ELISA technique. Bars indicate the mean ± standard deviation of groups. (****) indicates significant difference in relation to the saline, saponin, MPLA and ChimT groups (*p* < 0.00001). (&&&&) indicates significant difference in relation to the ChimT/Sap and ChimT/MPLA groups (*p* < 0.00001).

We examined the production of protein- and parasite-specific antibody, namely total IgG and IgG1 and IgG2a subclasses by recording optical density readings in ELISA in sera from immunized mice before and after infection. Mice immunized with ChimT/Sap and ChimT/MPLA, but not challenged with live parasite, had similar levels of total IgG that recognized ChimT and SLA, and which were significantly higher than the levels observed following immunization with ChimT alone (Figure 8A). The latter was capable of inducing IgG antibody at significant levels above the controls. Most of the antibody response induced by ChimT/Sap and ChimT/MPLA was of the IgG2a subclass, with little IgG1 antibody detected. All control groups showed low and similar anti-protein and anti-parasite IgG1 and IgG2a isotype levels. After infection, the antibody isotype production was maintained in the ChimT/Sap and ChimT/MPLA groups of immunized and infected mice, with again significantly high levels of anti-ChimT and anti-SLA IgG2a isotype, when compared to IgG1 values (Figure 8B). Now, the control mice produced high levels of total anti-SLA IgG, which was predominantly of the IgG1 subclass, with little IgG2a antibody detected.

### 3.4. ChimT/Sap and ChimT/MPLA Protect Mice from Parasite Infection

**Figure 9:**
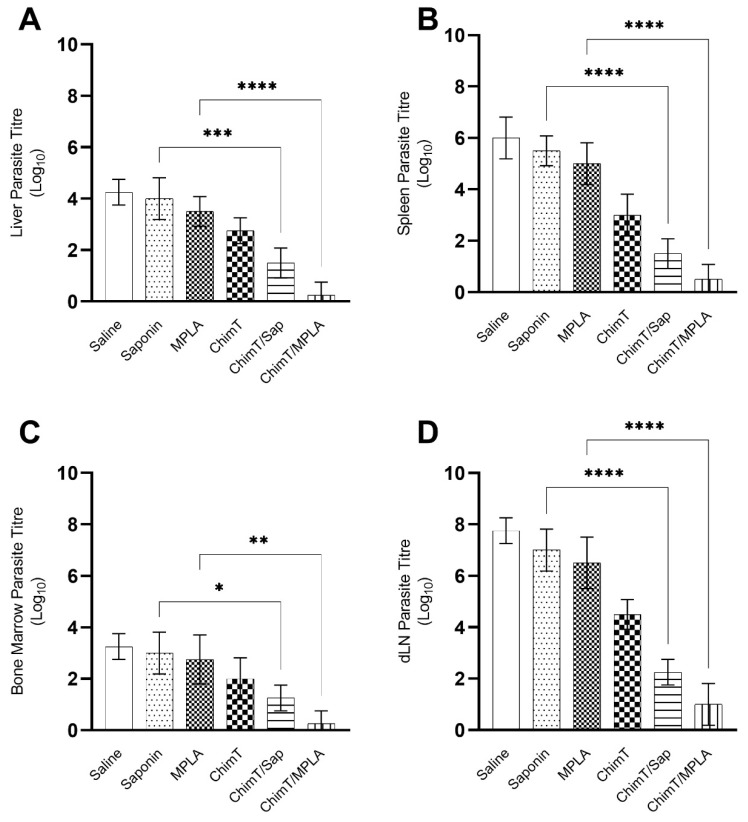
**Parasite load estimated by a limiting dilution technique.** Mice (*n* = 8 per group) received saline or were immunized with saponin, MPLA, ChimT, ChimT/Sap or ChimT/MPLA. Then, they were challenged with *L. infantum* promastigotes, and 45 days post-infection, their livers, spleens, bone marrow (BM) and draining lymph nodes (dLN) were collected to evaluate the parasite load through a limiting dilution technique. Data are expressed as the negative log of the titer adjusted per milligram of liver (**A**), spleen (**B**), BM (**C**) and dLN (**D**). Bars indicate the mean ± standard deviation of groups. (*), (**), (***) and (****) indicate significant difference in relation to the indicated groups by lines (*p* < 0.005, *p* < 0.001, *p* < 0.0001, and *p* < 0.00001, respectively).

**Figure 10:**
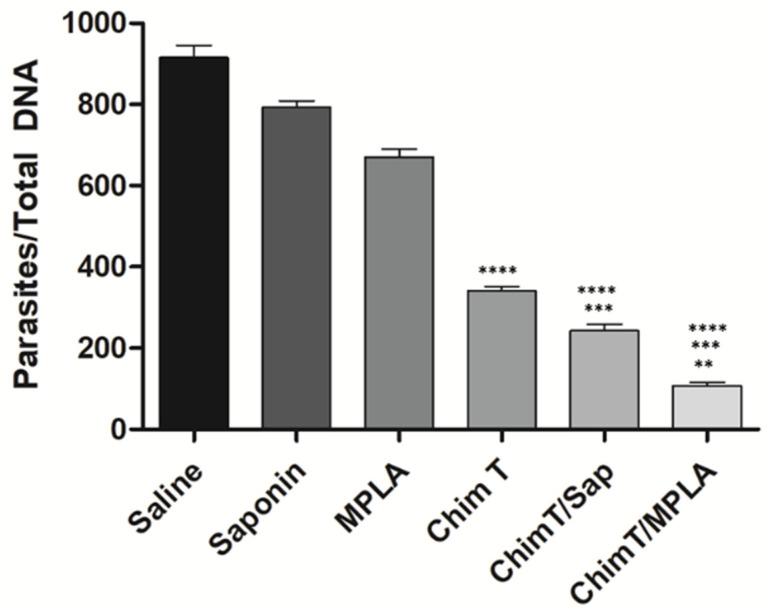
**Splenic parasitism evaluated by qPCR.** Mice (*n* = 8 per group) were vaccinated and later challenged with *L. infantum* promastigotes. Forty-five days post-infection, their spleens were collected and the parasite load was also evaluated by qPCR technique. Data are expressed as the number of parasites per 1000 nucleated cells. Bars indicate the mean plus standard deviation of the groups. (****) indicates significant difference in relation to the saline, saponin and MPLA groups (*p* < 0.00001). (***) indicates significant difference in relation to the ChimT group (*p* < 0.05). (**) indicates significant difference in relation to the ChimT/Sap group (*p* < 0.05).

The ability of ChimT, ChimT/Sap and ChimT/MPLA vaccines to protect mice against *L. infantum* infection was examined with a limiting dilution technique to quantify the parasite load at 45 days post-infection in the livers, spleens, bone marrow (BMs) and draining lymph nodes (dLNs) of the mice. The parasite load in the organs of ChimT/Sap and ChimT/MPLA immunized mice were significantly reduced compared to the control groups of mice (Figure 9). Reductions by an order of 2.5-, 4.0-, 1.7- and 4.7-log were found in the livers, spleens, BMs and dLNs, respectively, from the ChimT/Sap group of mice, as compared to the saponin control alone group, and reductions by an order of 3.2-, 4.5-, 2.5- and 5.5-log were found in the livers, spleens, BMs and dLNs. respectively, from the ChimT/MPLA group, when compared to mice receiving MPLA alone (Figure 9). Comparing the two vaccines, ChimT/MPLA reduced parasitism by an order of 1.2-, 1.0-, 1.0- and 1.3-log in the livers, spleens, BMs and dLNs, respectively, over the values found with ChimT/Sap-immunized mice. Splenic parasitism was also investigated by qPCR technique (Figure 10), and immunization with ChimT/Sap and ChimT/MPLA significantly reduced the parasite load in this organ by four- and approximately seven-fold, respectively, compared to data found in the control groups of mice and confirmed the data generated by the limiting dilution technique (Figure 9). However, ChimT alone did not appear to significantly reduce parasitism as judged by the limiting dilution technique (Figure 9) but did show an approximately two-fold reduction in splenic parasitism as judged by qPCR (Figure 10).

### 3.5. ChimT Stimulates IFN-γ Production from Human PBMCs

**Figure 11:**
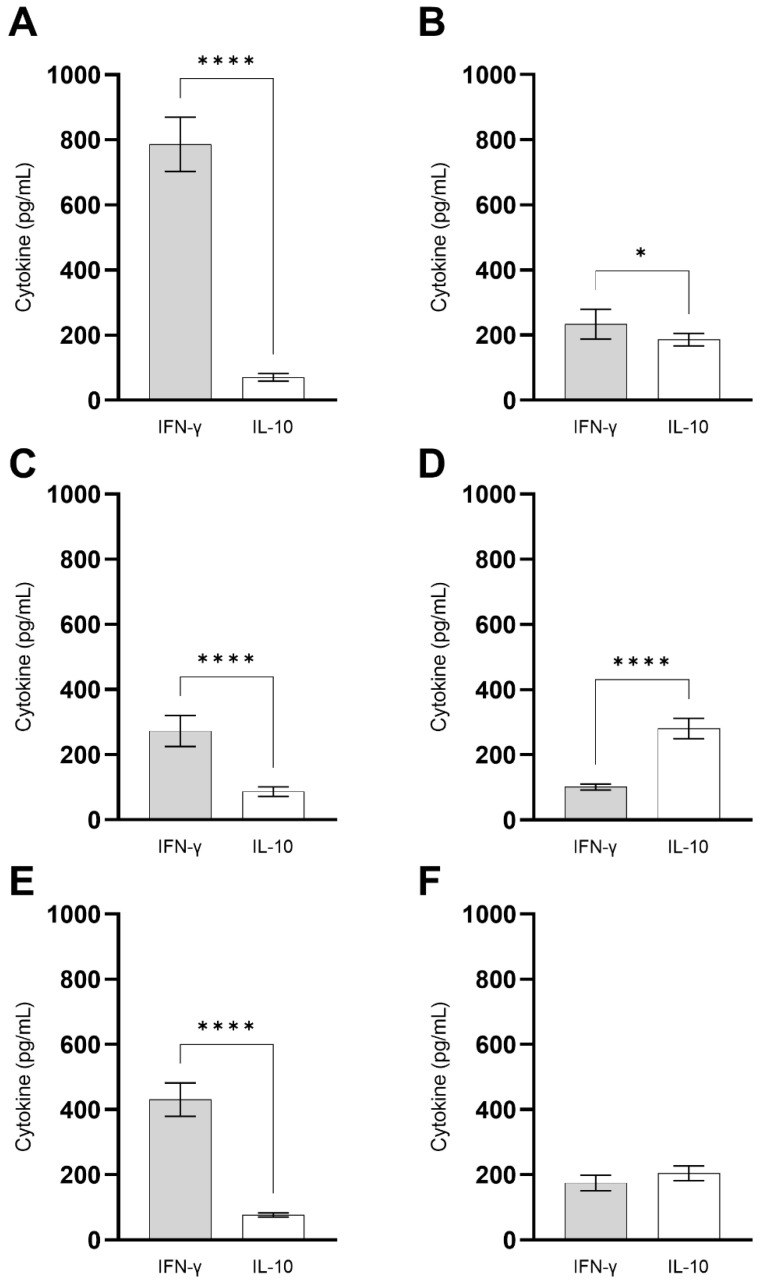
**Immunogenicity in human cell cultures.** PBMCs (1 × 10^7^ cells per mL) collected from healthy subjects (*n* = 6) and non-treated and treated visceral leishmaniasis (VL) patients (*n* = 6) were non-stimulated (medium) or stimulated with ChimT or SLA (10 and 25 µg/mL, respectively) for 5 days at 37 °C in 5% (*v*/*v*) CO_2_. The cell culture supernatants from healthy subjects (**A**,**B**) and from non-treated (**C**,**D**) and treated (**E**,**F**) VL patients were used to evaluate the IFN-γ and IL-10 production specific to ChimT (**A**,**C**,**E**) or SLA (**B**,**D**,**F**), which was measured by capture ELISA. Bars indicate the mean ± standard deviation of groups. (*) and (****) indicate significant difference in relation to the indicated groups by lines (*p* < 0.005 and *p* < 0.00001, respectively).

PBMCs were isolated from blood samples from treated and untreated VL patients and from healthy individuals, and they were stimulated in vitro with the recombinant ChimT protein and SLA, and the levels of IFN-γ and IL-10 cytokines were quantified in the supernatants. Stimulation with ChimT induced significantly higher levels of IFN-γ in cells from healthy subjects (Figure 11A) when compared to values found in the cell cultures from VL patients (Figure 11C,E) as well as when compared to values obtained using SLA as stimulus (Figure 11B). Between VL patients, those treated (Figure 11E) showed approximately two-fold higher levels of IFN-γ compared to values found in cell cultures from untreated patients (Figure 11C). Regarding IL-10 production, higher levels of this cytokine were found in the cell supernatants from untreated VL patients after stimulus using SLA (Figure 11D) compared to values from treated patients (Figure 11F) and with using ChimT as cellular stimulus (Figure 11C,E). In general, ChimT induced higher IFN-γ and lower IL-10 levels when compared to values obtained using SLA as stimulus in all experimental groups.

## 4. Discussion

Considerable efforts have been made to develop a vaccine to protect against VL, and although a few vaccines are licensed for canine use, no vaccine exists for use in humans [33]. New and more effective vaccines are needed to improve the clinical condition of infected dogs and to interrupt the sand fly vector transmission cycle and to protect humans where VL is an anthroponosis [34]. In the present study, we examined the potential of a new chimeric protein, ChimT, to protect a murine model against *L. infantum* infection and whether it could also stimulate immune cells from healthy individuals and patients with VL, before and after treatment. The key findings from our study were that (1) immunization of mice with ChimT, with saponin or MPLA adjuvants generated a specific Th1-type immune response with proliferation of T cells and protected them against infection with *L.* *infantum* and (2) ChimT stimulated the production by ex vivo human immune cells of high levels of IFN-γ and low levels of IL-10, cardinal signals of a Th1-type immune profile.

*Leishmania* spp. are obligate intracellular parasites that can reside within immune cells, such as monocytes/macrophages, dendritic cells and neutrophils, among others, and they possess various strategies to avoid the parasiticidal capacity of the host immune system [35]. After initial inoculum of *Leishmania* by infected sand flies, the promastigotes are phagocytosed by such cells, and two outcomes are possible: (1) promastigotes become amastigotes by simple fission and they can multiply within the cell until the cell lyses and releases parasites into the blood and interstitial space or (2) internalized parasites are killed by the immune cells [36]. Since amastigotes display fewer antigens than promastigotes, vaccines that direct the host immune response towards specific amastigote antigens could protect against infection [29]. In our current study, the LiHyp1 [37], LiHyV [38], LiHyC [39] and LiHyG [30] proteins that we selected are all found in the *L. infantum* amastigote stage and a chimeric vaccine was developed using the main T-cell epitopes from each protein. The rationale for this approach is supported by previous studies using single, recombinant parasite proteins and chimeric proteins containing antigens expressed in the promastigote stage of the parasites [22,40].

The development of a polarized and specific Th1-type T-cell immune response is required for protection against *Leishmania*, and the presentation of T-cell epitopes derived from parasite immunogenic proteins through the MHC I and MHC II pathways is believed necessary to induce immunological and parasitological protection [41]. Effective vaccines against leishmaniasis will most likely require adjuvants to increase immunogenicity [42,43] and to provide an added benefit in reducing both the required antigen dose and administered number of doses [44,45]. However, the choice of adjuvants for *Leishmania* vaccines is limited to a few that are appropriate for use in dogs and humans. Saponins have been extensively used as adjuvants in leishmaniasis vaccines, successfully triggering a Th1-type response and activation of both CD4^+^ and CD8^+^ T cell-subtypes in immunized dogs [46] and in a mouse model [25]. However, saponins show some toxicity towards mammals, and their use is not generally authorized for humans, except for more purified and expensive fractions [47]. MPLA has been shown to stimulate a polarized Th1-type immune response in immunized mice, with the concomitant production of IL-2, TNF-α and IFN-γ cytokines and expression of costimulatory molecules [48,49]. MPLA is a clinically proven adjuvant for humans with low toxicity and the ability to efficiently stimulate T-cell-mediated immune responses, making it an appropriate candidate for use in vaccines against intracellular pathogens such as *Leishmania* [50]. In the present study, although ChimT tested alone induced a weak Th1-type response, it was unable to protect immunized mice from parasite infection. The refractory response of ChimT was overcome by using either both saponin or MPLA and resulted in robust Th1-type immune responses and significant reductions in parasite loads in infected mice. Although the levels of protection were similar, MPLA is preferred as an adjuvant, mainly because mice immunized with ChimT/Sap showed transient edema at the inoculation site, an adverse effect not observed with ChimT/MPLA. The use of saponin could be viewed as a limitation of the current study, although it was useful to validate the hypothesis that ChimT could generate a protective immune response. Future pre-clinical studies could test the efficacy of human compatible saponin fractions, as used for example in Novavax’s NVX-CoV2373 protein-based COVID vaccine, as well as many other adjuvants. These studies would be critical, as adjuvant matching may be necessary, depending on their properties and synergism with ChimT. Moreover, incorporation of ChimT and MPLA into liposomes, which are often used as carriers for this adjuvant, could be one potential method to enhance immunogenicity.

In our study, ChimT was constructed with CD4^+^ and CD8^+^ T-cell-specific epitopes, derived from distinct parasite proteins, in an attempt to produce an antigen able to induce better immunogenicity, by production of cytokines, such as IFN-γ, IL-2, IL-12, GM-CSF and TNF-α, among others [51]. Our rationale was based on the facts that the presence of CD4^+^ T-cell epitopes could induce the immune response in the initial phase of infection, while CD8^+^ T-cell epitopes could help to guarantee the long-term immunity against parasite infection [52]. In this respect, studies have shown that protection against VL is associated with the production of Th1-type cytokines by both T-cell subtypes [53]. We showed directly by flow cytometry and indirectly by inhibition experiments with monoclonal antibodies that both T-cell subtypes were important to produce IFN-γ in mice immunized with ChimT/Sap and ChimT/MPLA, as well as a positive proliferative response when ChimT was used to stimulate murine spleen cells in vitro. Conversely, anti-inflammatory cytokines, such as IL-4, IL-5, IL-10 and IL-13, among others, seem to act to regulate the Th1-type response and favoring the development of infection [54]. Their role as molecules that modulate the Th1-type immune response and control the exacerbated production of pro-inflammatory cytokines is also a consideration [54,55]. In our study, spleen cells of ChimT/Sap or ChimT/MPLA immunized mice produced low levels of IL-4 and IL-10 cytokines in response to protein and parasite stimuli. The low levels of these cytokines were observed in spleen cell cultures before and after challenge, which supports the conclusion that a pro-inflammatory response profile is established in this animal model.

We also observed the increased production of nitrite, a proxy for nitric oxide, in stimulated spleen cells from mice immunized with ChimT/Sap or ChimT/MPLA and subsequently infected. Production of this anti-leishmanial molecule can be considered as a biomarker of immunity against *Leishmania* infection. The presence of activated macrophages, which produce nitric oxide and reactive oxygen species, is associated with the development of a Th1-type response via production of cytokines such as IFN-γ and TNF-α. Our data are supported by other studies, where similar results were found when immunogens induced high anti-leishmanial nitrite production [56,57,58]. By contrast, non-immunized control mice had low nitrite levels, which correlated with higher parasite loads found in their internal organs. Immunization with ChimT alone did induce some nitrite production, but this was insignificant compared to the levels induced by the ChimT-adjuvant vaccines. It is possible that there was low activation of macrophages with ChimT alone, and this demonstrates the importance of the exogenous adjuvants in stimulating immune activity biomarkers.

Vaccine efficacy was evaluated by determination of the parasite load in distinct organs of infected mice, and this is based on a study of primary and secondary sites of infection by *Leishmania* spp. [59]. The dLN and liver are considered primary organs of infection, where high parasitism is found in the initial periods of infection, while organs such as the spleen and BM are characterized as secondary sites of infection, when high parasitism is found usually in the later period of infection [60]. In the current study, we observed a slight reduction in parasite load in the organs of mice immunized with ChimT alone. Thus, despite the Th1-type immune response found in these animals before and after infection, the protein alone was unable to provide significant protection against infection. However, the addition of adjuvants saponin or MPLA to ChimT stimulated significant reductions in organ parasitism levels. Similar results have been obtained with recombinant antigens plus adjuvants in the murine VL model. Although mice are widely useful in obtaining detailed analyses of immunological and parasitological responses and contribute to the selection of possible vaccine candidates, a limitation of the current study is that parasitological data obtained in rodents cannot be extrapolated to other mammals. Thus, further investigations of the prophylactic potential of ChimT against *Leishmania* infection should be conducted in dogs and humans. As a step to understanding the immunogenicity of ChimT potentially in humans, we did show that ChimT could induce an ex vivo Th1-type response in human PBMC cultures, with high levels of IFN-γ and low levels of IL-10 produced by ChimT-stimulated cells from healthy individuals and VL-treated patients. When SLA was used as stimulus, lower levels of IFN-γ and higher levels of IL-10 were observed. Thus, our preliminary data could be considered as a proof of concept of the potential capacity of ChimT to stimulate immunity in humans [61]. However, additional studies are necessary to resolve these questions.

## 5. Conclusions

Experimental recombinant chimeric protein-based vaccines containing T-cell epitopes from a variety of different *Leishmania* proteins have been shown to induce protective immune responses against live infection of animal models [25]. In the current study, we showed that a new recombinant chimeric molecule, ChimT, composed of T-cell epitopes derived from *Leishmania* amastigote proteins and administered with exogenous adjuvants induced a Th1-type immune response and protection against murine VL. Hypothetically, ChimT could induce better immune and protective immune responses than previous chimeric candidates since it contains epitopes from proteins expressed by *Leishmania* amastigotes, which are responsible for the development of infection and active disease. However, a limitation of the current study was that we did not test this hypothesis by comparing the immunogenicity of these different chimeric candidate vaccines directly with each other. Regardless, ChimT/adjuvant immunization generated a pro-inflammatory immune profile based on high levels of IFN-γ, IL-2, IL-12, GM-CSF and TNF-α cytokines and induced the production of anti-leishmanial nitrite and IgG2a antibodies, which resulted in significantly reduced parasite loads in mouse internal organs [62,63]. Our preliminary study suggests that the T-cell-epitope-based chimeric protein ChimT is a promising candidate for further evaluation with comparison alongside other candidate chimeric vaccines in other mammalian hosts to protect against VL.

## Figures and Tables

**Table 1:** Characteristics of ChimT T-cell chimeric protein.

ChimT Sequence	KKKKG-LFVNQKLVGTIADVRSYEK (XP_001470432.1; LiHyC)-GKKG-YIMSGPARYVYFHMVLPVEAQ (XP_001468941.1; LiHyp1)-GKKKG-GVCVANTNVAAGAHTAALAAAVCVV (XP_001462854.1; LiHyV)-GKKKG-SLFVLYMYVTCRGGYTYLQL (XP_001467126.1; LiHyG)-GKKKK
Physical–chemical characteristics	113 amino acid residues
	Molecular weight of 11.9 kDa
	Isoelectric point of 10.71
	Instability index of 6.09
	Aliphatic index of 93.27
	Grand average of hydropathicity (GRAVY) of 0.019

Amino acid sequence of the chimeric protein ChimT and identification of the origin proteins and the physical–chemical characteristics of the recombinant protein.

## Supplementary Materials

The following supporting information can be downloaded at: https://www.mdpi.com/article/10.3390/vaccines10071146/s1, Figure S1. Representative plots of gating strategy to evaluate IFN-γ, TNF-α, IL-2 and IL-10-producing T cells. The figure shows the control cultures (negative and positive cultures). The positive culture was stimulated with Phorbol 12-Myristate 13-Acetate (PMA-5 ng/mL) and ionomycin (1 mg/mL).
