# Supplementary material for: A Recombinant Chimeric Protein-Based Vaccine Containing T-Cell Epitopes from Amastigote Proteins and Combined with Distinct Adjuvants, Induces Immunogenicity and Protection against Leishmania infantum Infection
Source: Vaccines (Basel). 2022 Jul 19;10(7):1146. doi: 10.3390/vaccines10071146 (PMC9317424; doi:10.3390/vaccines10071146)
Supplement: Supplementary file 1 [file vaccines-10-01146-s001.zip › vaccines-1805840-supplementary.pdf]

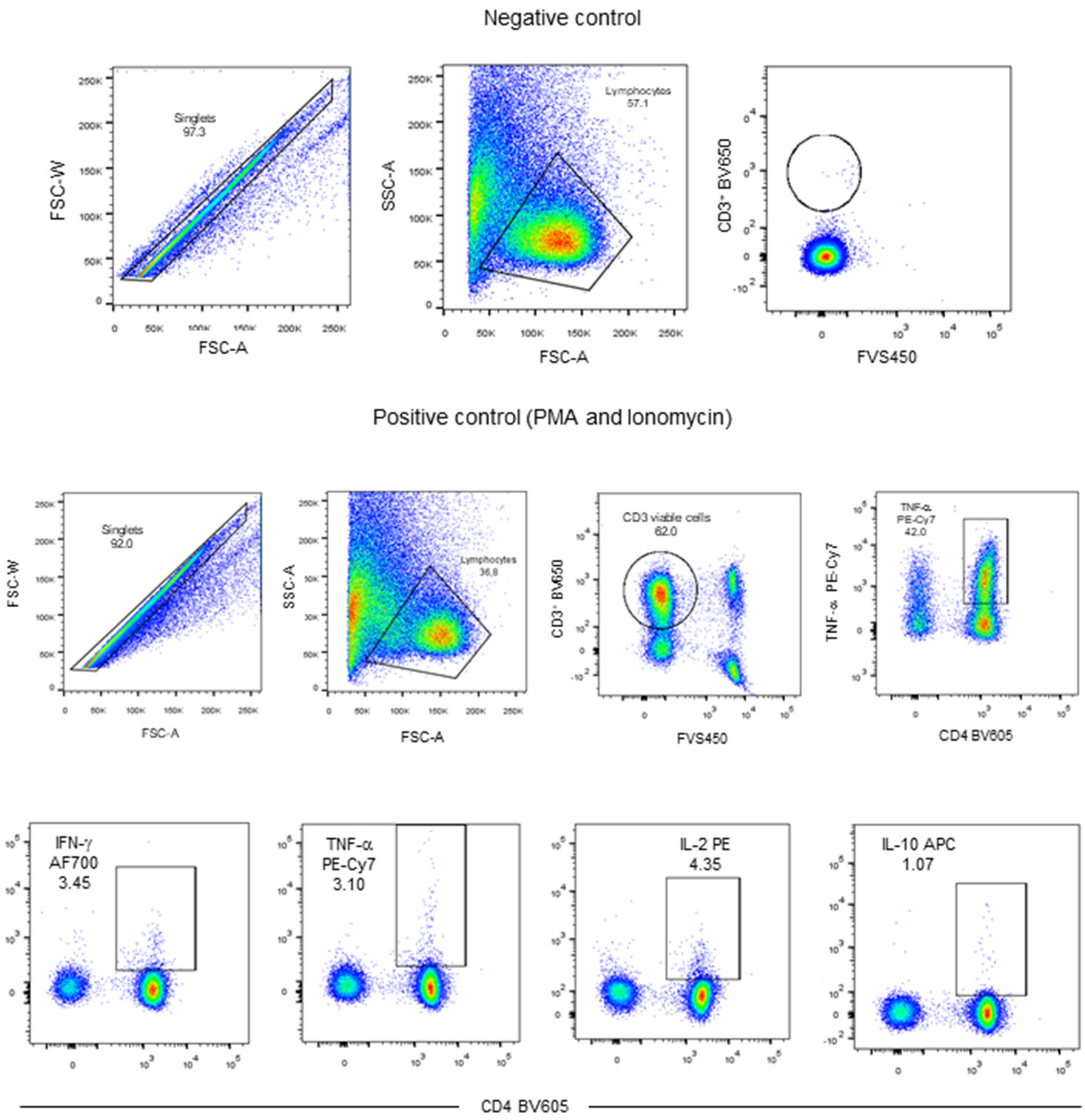

Figure S1. Representative plots of gating strategy to evaluate IFN-γ, TNF-α, IL-2 and IL-10-producing T cells. The figure shows the control cultures (negative and positive cultures). The positive culture was stimulated with Phorbol 12-Myristate 13-Acetate (PMA-5 ng/mL) and ionomycin (1 mg/mL).
